# Efficient removal of ciprofloxacin from aqueous solution using Zn–C battery derived graphene oxide enhanced by hydrogen bonding, electrostatic and π-π interaction

**DOI:** 10.1016/j.heliyon.2024.e33317

**Published:** 2024-06-21

**Authors:** Sabina Yasmin, Md Golam Azam, Md Sanwar Hossain, Umme Sarmeen Akhtar, Md Humayun Kabir

**Affiliations:** a Institute of National Analytical Research and Service (INARS), Bangladesh Council of Scientific and Industrial Research (BCSIR), Dhanmondi, Dhaka, 1205, Bangladesh; b Institute of Glass and Ceramic Research and Testing (IGCRT), Bangladesh Council of Scientific and Industrial Research (BCSIR), Dhanmondi, Dhaka, 1205, Bangladesh

**Keywords:** Zn-C battery, Graphene oxide, Density functional theory (DFT), Ciprofloxacin, Adsorption, Kinetics

## Abstract

In this study, graphene oxide (GO) derived from waste Zinc–Carbon (Zn–C) batteries was proposed for the efficient removal of antibiotics from the aqueous solution. Ciprofloxacin (CIP) antibiotic was selected as a typical contaminants. GO was prepared via an economical and environment-friendly route by using carbon rods from waste Zn–C batteries as the precursor. Characterization techniques were applied to determine the properties of as prepared GO. Effects of pH, contact time, and adsorbent dose on the adsorption were explored, and an optimum condition was established. Adsorption equilibrium was established in just 20 min for maximum removal of CIP (99.0%) at pH 5.7 for the adsorbent dose of 20 mg L^−1^ and at the initial concentration of CIP 2.0 mg L^−1^. The rapid and efficient removal of CIP was greatly influenced by the electrostatic attractions, pi-pi interactions and hydrogen bonding on the surface and edge of GO which was also proved by density functional theory (DFT). Langmuir model showed the best fit among the isotherm models and the calculated maximum adsorption capacity (q_m_) was 419.62 mg g^−1^ at 30°C. The kinetic studies also revealed that the adsorption process followed the pseudo-second-order model. The endothermic and spontaneous nature of adsorption was evaluated in thermodynamic studies.

## Highlights

• Preparation of Graphene Oxide (GO) from waste Zn–C battery to remove CIP.
• Prepared GO shows removal efficiency of 99 % for CIP within very short time.
• Adsorption kinetics, isotherm, and thermodynamic studies were performed.
• Adsorption mechanism investigated supporting with DFT calculation.

## Graphical abstract

**Figure:**
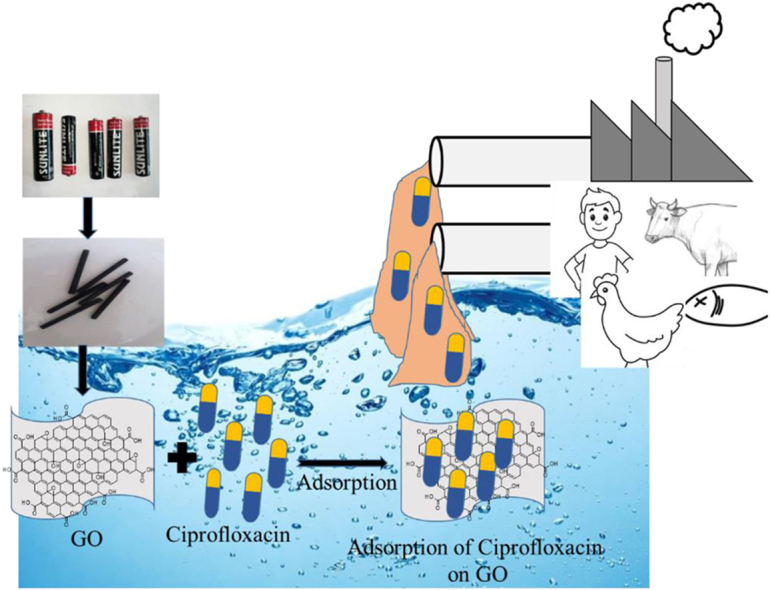

## 1 Introduction

In recent years, the use of antibiotics has increased dramatically. Between 2000 and 2015, the growth in the global antibiotic consumption was estimated to be 65% (from 21.1 to 34.8 billion Defined Daily Doses), and by 2030, it was predicted to increase by another 200% [1,2]. Antibiotics are largely used for infectious diseases in humans and animals, and for the growth of animals and fish in farms [3,4]. Due to the incomplete absorption in the gut, antibiotics come out with the excretion of humans and animals, and enter the sewage water [5,6]. Sewage water and pharmaceutical wastewater have been reported to have antibiotic pollutants in the level of μg L^⁻1^ to mg L^⁻1^ [[7], [8], [9], [10], [11]]. Untreated discharge of wastewater introduces antibiotics into the waterbodies of environment. Since many antibiotics are not easily degraded in the environment or have a long half-life, they persist in the aqueous system, and deposit in sludge and soil [[12], [13], [14]]. Via irrigation and the exposure of fish to the contaminated water, antibiotics can enter into the food chain [15,16]. The existence of antibiotics in the environment induces antibiotic resistant genes in the bacteria which can lead to untreatable diseases and infections in humans and other animals [[17], [18], [19]]. Antibiotic resistant superbugs killed 1.27 million people in 2019 [20]. Besides, antibiotic residues in food can alter the microbiome community in the gut of humans and animals, leading to poor-health and chronic diseases [21]. Persistence of antibiotics in the waterbodies and terrestrial areas negatively affect the ecosystem [22]. It is obvious that, proper treatment of municipal and pharmaceutical wastewater is of utmost importance to prevent antibiotic pollution.

CIP is a second-generation synthetic fluoroquinolone antibiotic, that is largely used in the treatment of a wide range of both gram-positive and gram-negative bacterial diseases [[23], [24], [25]]. Due to its incomplete absorption in the applied subjects and slow degradation, it can persist in the environment—several studies have shown its presence in municipal effluent, hospital effluent, pharmaceutical effluent, surface water, soil, fishes, vegetables, and plants above the antibiotic resistance and eco-toxicologically predicted no-effect concentration [7,[26], [27], [28], [29], [30], [31]]. Therefore, CIP was chosen as a target pollutant in this study.

Several methods have been developed for the removal of antibiotics from aqueous system to date, such as advanced oxidation [32], enzymatic reaction [33], photocatalytic degradation [34,35], Biodegradation [36], membrane process [37], nano-filtration [38], nanomaterials [39,40] and adsorption [[41], [42], [43]]. These methods suffer from different difficulties, like insufficient removal, toxic byproduct formation, sludge formation, disposal management, and high operational costs and energy requirements. In the removal of antibiotics, adsorption has been proven to be a promising method due to its superior removal efficiency, simple design, less toxic intermediate formation, regeneration of materials, and low cost [44]. Even though the adsorption method has numerous advantages, this process is limited by lengthy operations, high operation costs, low efficiency of the adsorbent, and scarcity of the adsorbent source. These limitations can be overcome by developing an adsorbent with a significant number of functional group, large surface area, and pi-electron rings for fast and effective adsorption, and the adsorbent can be derived from waste Zn–C battery to solve the scarcity of adsorbent sources, minimize the cost of adsorbent, and solve the environmental problems. Graphene is an atomic-scale hexagonal lattice consisting of carbon atoms. Applications of graphene and its derivatives in different fields such as material technology, energy, medical science, and environmental remediation have been extensively investigated owing to their remarkable properties in terms of versatility, optical transparency, electrical conductivity, mechanical strength, and thermal conductivity [[45], [46], [47], [48]]. In GO, both surfaces of graphene are loaded with oxygen-containing functional groups [49]. GO and its derivatives have garnered attention from researchers in the fields of adsorption by virtue of their large surface area, chemical tunability, outstanding dispersion properties, and functional groups [[50], [51], [52], [53]]. For these properties, GO based adsorbent was used to remove antibiotics from aqueous environment [54]. GO can adsorb antibiotics from aqueous system by electrostatic (π-π and ionic) interaction, hydrogen bonding, and hydrophobic interaction [55,56].

Graphite is a naturally occurring solid substance consisting of sp^2^ hybridized carbon atoms arranged in hexagonal lattices [53,57]. Graphene oxide (GO) is the oxidized derivative of graphene, which possesses numerous oxyginated functional groups [58]. The preparation of GO requires high quality graphite as a raw material, however, natural resources of graphite are limited, and its synthesis is relatively expensive [59]. For these reasons, different approaches have been made earlier to find economical and sustainable raw materials for mass production of GO [60].

Zn–C batteries are used worldwide in different devices such as toys, flashlights, clocks, etc, [61]. A carbon or graphite rod is present in the center of the Zn–C battery and serves as a positive electrode. The electrode is surrounded by a combination of manganese dioxide and carbon powder. Being non-rechargeable, each year, thousands of these batteries are discarded globally [3]. The chemicals and metals presented in these discarded batteries break down over time and leak into the environment. Generally, carbon rod of used Zn–C batteries do not undergo corrosion/change, hence, these can be employed for the synthesis of GO. Reusing of these carbon rods in preparing GO offers a fantastic waste management solution and opens up a new source of simple raw materials for GO production. In our previous studies, this material has been used to remove lead and azithromycin from aqueous media where the adsorption capacity was not so high [51,62]. More study with different antibiotics is needed to find out the best fitness of this material as a versatile adsorbent. Therefore, the current study attempted to evaluate the adsorption capacity and mechanism of Zn–C batteries derived GO for CIP removal from aqueous solution.

In this study, GO was synthesize from carbon rods of waste Zn–C batteries and applied it as an adsorbent to remove CIP from an aqueous system. The aim of this study were: (1) to assess the adsorption capacity of GO on CIP (2) to describe the adsorption mechanism by experiment and density functional theory. The concentration of CIP before and after adsorption in the samples were determined by liquid chromatography–tandem mass spectrometry (LC-MS-MS). We found that Zn–C battery derived GO adsorbs CIP rapidly and shows the best adsorption capacity (419.62 mg g^−1^) mainly through electrostatic interaction, H-bonding, and pi-pi interaction, which is in agreement with the results of density functional calculations. So the cost effective waste product derived GO can rapidly and efficiently remove the CIP from the aqueous media.

## 2 Materials and methods

### 2.1 Chemicals

Ciprofloxacin VETRANAL™ (C_17_H_18_FN_3_O_3_) (≥98%, analytical standard, CAS: 85721-33-1) was obtained from SIGMA-ALDRICH, Switzerland. Potassium permanganate (KMnO_4_) (≥99.0%, CAS: 7722-64-7) and Sulfuric acid (H_2_SO_4_) (95–97%, reagent grade, CAS: 7664-93-9) were purchased from Scharlau, Spain. Phosphoric acid (H_3_PO_4_) (85 wt% in H_2_O, CAS: 7664-38-2) was obtained from JANSSEN CHEMICA, Belgium. Ethanol (C_2_H_5_OH) (98%, CAS: 64-17-5) and Hydrochloride acid (HCl) (37%, Extra pure, CAS: 7647-01-0) were obtained from AppliChem, Germany. Hydrogen per oxide (H_2_O_2_) (30%, CAS: 7722-84-1) was collected from Scharlau, Spain. Ultrapure deionized (DI) water (18MΩ-cm) was used in the preparation of all the aqueous solutions.

### 2.2 Instruments

Microstructure, surface morphology, and composition of GO were studied using Transmission Electron Microscopy (TEM; JOEL JEM-2100F, operated at 200 kV) and Field Emission Scanning Electron Microscopy equipped with Energy Dispersive X-ray Spectroscopy (FESEM-EDX; JEOL-JSM-7610F, 0.1kV–30kV). The XRD patterns were studied using a Cu-Kα source (λ = 1.5406 Å) in a Rigaku (Japan) instrument. FTIR spectra were obtained using the SHIMADZU IRAffinity-1 (Japan) spectrometer. Raman analysis was performed at an excitation wavelength of 514 nm using HORIBA macro RAM Raman Spectrometer. To determine the surface area and pore volume, Nitrogen adsorption-desorption isotherms were studied at 196 °C (77K) with the help of a PMI BET sorptometer (BET-201-A), degassing in vacuum was performed at 40 °C before each measurement. The CIP concentration was measured in an Agilent LC module (1290 Infinity II) linked with a triple quadruple mass spectrometer (6420 LC/TQ). For analytes separation, a ZORBAX RRHD Eclipse Plus C18 (2.1 × 100 mm, 1.8 mm particle size) was employed. The mobile phase consisted of two segments, one was 0.1% formic acid (in water), and the other was acetonitrile. The linear isocratic mobile phase was run using equal volume from both segments, with a 0.3 mL/min total flow rate. Positive electron spray ionization mode (ESI^+^) was employed for the analysis. Multiple Reaction Monitoring (MRM) transitions and Mass spectrometry parameters for the LC-MS/MS analysis of CIP was as two transition of molecular weight from 332 to 314 and 231 as quantifier and qualifier, respectively, for both transition dwell voltage was 200 V fragmantor 110 V, collision energy 20 and 35 eV for the quantifier and qualifier, respectively.

### 2.3 Graphite recovery

**Scheme 1:**
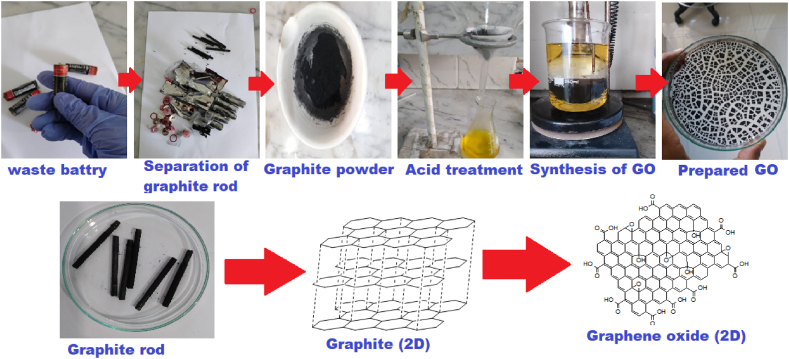
Synthesis of GO from waste Zn–C battery.

Spent Zn–C batteries were collected from several houses and market places. Carbon rods were pulled apart using pliers. The electrodes were wiped clean with paper and washed with DI water several times to get rid of adherent MnO_2_, NH_4_Cl, and carbon. After that, these were dried in an oven at 60 °C for 24 h and crushed into a fine powder by mortar and pestle. The dried powder was then treated with HCl and HNO_3_ (3:1) for 2 h at 60 °C (Scheme 1). Later, it was centrifuged and washed with DI water several times. After that, the treated powder was then dried in an oven for 48 h at 60 °C.

### 2.4 Synthesis of graphene oxide

An improved Hummers' method was applied to prepare GO [63,64]. In brief, 1.0 g recovered graphite powder from Zn–C batteries was added to a solution of H_2_SO_4_ and H_3_PO_4_ (ratio 9:1 v/v) with continuous stirring, followed by addition of 6 g KMnO_4_. The mixture was heated at 50 °C for 12 h with constant stirring until it turned into dark green. The reaction mixture was then cooled to room temperature and poured in 400.0 g ice and 3 mL 30% H_2_O_2_ for reaction to stop and for elimination of excess KMnO_4_. The mixture was then centrifuged at 3500 rpm. The resulting precipitate was first washed with DI water, then with 30% HCl to remove metallic impurities, then rewashed with DI water to remove chloride, and then with ethanol. After that, as prepared, GO was dried at 45°C in a vacuum drying oven for 48h (Scheme 1).

### 2.5 Adsorption studies

100 ppm stock solution of CIP in methanol was prepared by taking 10.0 mg of powdered standard in a 100 mL volumetric flask and then adding methanol up to the mark. Working standard solution was prepared by taking required volume of stock solution in others volumetric flasks and diluted with same solvent to prepare required working solution. Which was later used to prepare aqueous solutions at CIP of different concentrations. CIP solution (40 mL) was poured into 100 mL Erlenmeyer flasks, and the pH was adjusted using HCl and NaOH, The adjusted pH was monitored using a pH digital Multimeter-parameter (HQ40d, HACH, USA). After that, the required amount of GO was added, and the mixture was shaken at 230 rpm in an orbital shaker (SSL-1, Stuart, Germany). It was centrifuged at (Velocity 18R, Australia) and filtered through a 0.45 μm CHROMAFIL® Xtra syringe filter after required period of shaking. The concentrations of CIP solutions were determined by a LC-MS/MS (Agilent 1290 TQ 6420) instrument before and after adsorption. The optimal adsorption conditions were investigated by varying the pH (2-10) of the CIP solution, the adsorbent dose (8–125 mg L^−1^), and the contact time (5–80 min), which were later applied in experiments for isotherm and kinetic studies. In kinetic investigations, contact time was variable, and in isotherm studies, the initial concentration of the CIP solution was also variable. Thermodynamic experiments were carried out at three distinct temperatures (30, 40, and 50 °C). The removal percentage, adsorbed amount at time, t (q_t_) and at equilibrium time, (q_e_), were calculated by using the following Equations (1)–(3), respectively [65]**.**

(1) $$\text{Removal}\;\text{percentage}=\frac{C_{0}-C_{t}}{C_{0}}\times 100\%$$

(2) $$q_{t}=\frac{C_{0}-C_{t}}{M}\times V$$

(3) $$q_{e}=\frac{C_{0}-C_{e}}{M}\times V$$

Where *C*0, *C*t, and *C*e stand for the initial, at time t, and equilibrium concentrations of TCs solutions, measured in mg/L. M represents the amount of adsorbent, measured in g. V signifies the volume of TCs solutions measured in L.

### 2.6 Statistical analysis

The analysis of all experimental data were studied by performing triplicate measurements\and computing the average outcomes. The regression coefficient (R^2^) and constant values of isotherms (Langmuir, Freundlich, and Temkin) and kinetic models (pseudo-first-order, pseudo-second-order, and intra-particle diffusion) were examined by using the statistical program of Sigma plot 10.0 [66].

## 3 Results and discussion

### 3.1 FESEM-EDX and TEM analysis

**Fig. 1:**
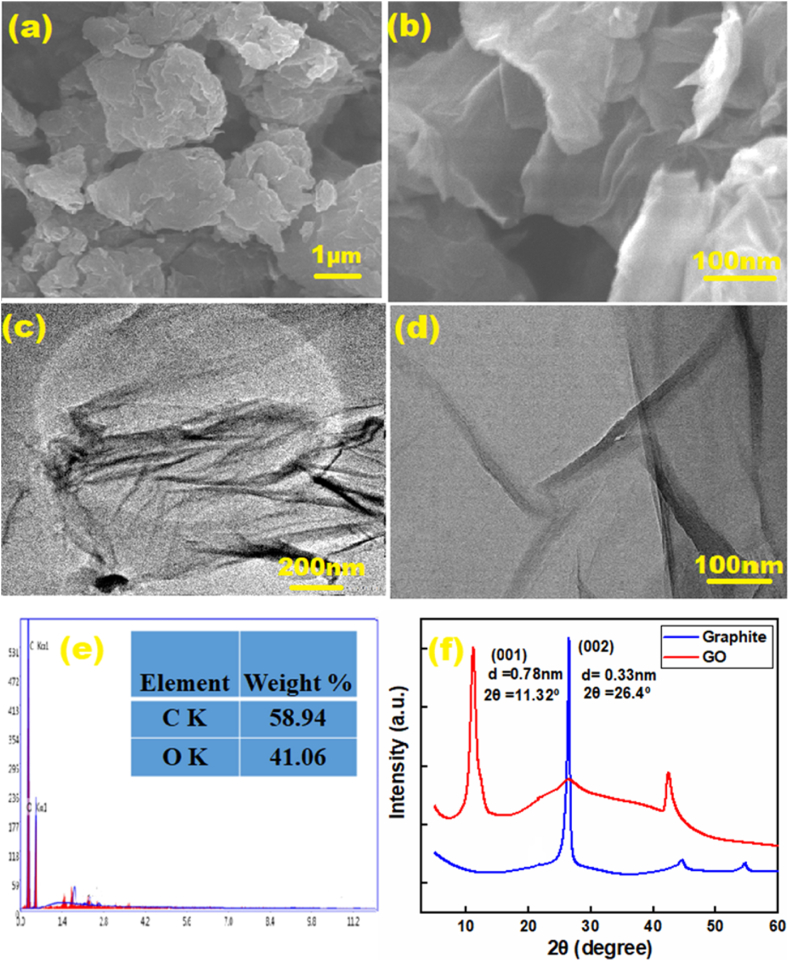
FESEM image (a–b), TEM image (c–d), EDX spectra (e), and XRD pattern (f) of GO.

To observe the morphology of GO, FESEM and TEM images were taken (shown in Fig. 1(a) and (b-d)). The FESEM image exposes the highly porous, layered, and stacked structure of GO, such structural properties of an adsorbent are associated with enhanced adsorption capacity due to greater exposure of surface area.

The TEM image gives a different perspective on the morphology of GO, it reveals that the structure of as prepared GO consists of well-defined multilayers that are wrinkled in a randomly distributed fashion and folded in some areas.

The EDX analysis (Fig. 1(e)) showed the carbon oxygen (C/O) ratios in of the as prepared GO was 1.42. Similar morphology and C/O ratio GO have been reported in several reports, which further ensures the successful preparation of GO [[67], [68], [69]].

### 3.2 XRD analysis

Fig. 1(f) shows the XRD pattern of graphite powder and GO. Graphite powder gives the diffraction peak at 2θ = 26.4°, corresponding to the crystal plane (002), with a d-spacing of 0.33 nm calculated from Bragg's equation [70,71]. In the pattern of GO, the peak appeared at 2θ = 11.32° which corresponds to crystal plane (001) [72], and the d-spacing was calculated to be 0.78 nm, these are characteristics of GO as supported by previous studies [[64], [65], [66]]. The increase in d-spacing between the layers in GO compared to graphite is due to the functional groups introduced into the carbon layers by oxidation [73].

### 3.3 FTIR analysis

**Fig. 2:**
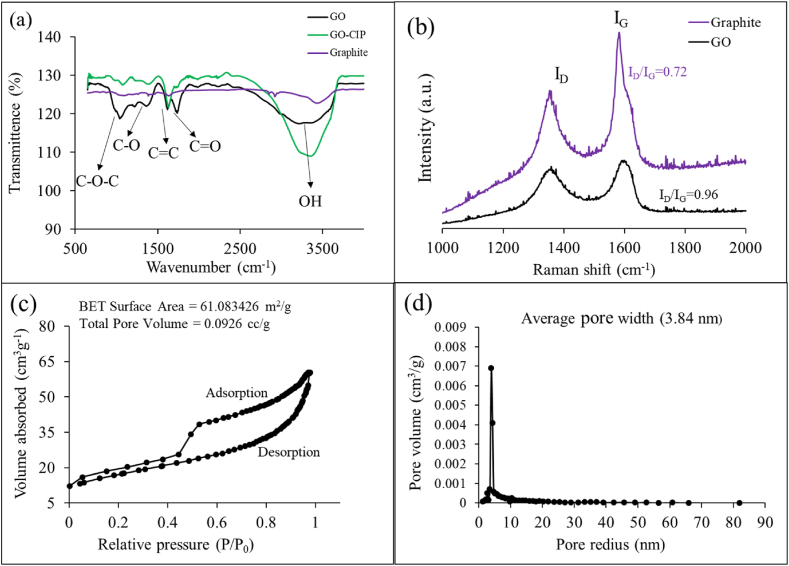
FTIR spectra of graphite, GO before and after adsorbing CIP (a), Raman spectra of graphite and GO (b), and BET analysis (c–d) of GO.

Functional groups of an adsorbent actively participate in adsorption, therefore, studies of functional groups are vital to understand the adsorption mechanism. The FTIR spectra of graphite and GO (before and after adsorption of CIP) have been shown in Fig. 2(a). The absence of any notable peak in the FTIR spectra graphite suggests that graphite is chemically inert. While, FTIR spectra of GO exhibited various functional groups. The broad and strong peak observed around 3345 cm^−1^ indicates the presence of O–H stretching from hydroxyl and carboxyl groups [74]. The peak observed at 1715 cm^−1^ refers to C=O stretching from carboxyl groups [75]. Appearance of the peak at 1585 cm^−1^ evinces the presence of C=C stretching from framework of GO [76]. The strong peaks appearing at 1010 cm^−1^ is characteristic of C–*O*–C stretching from the epoxy group [[77], [78], [79]]. And, many oxygenated groups such as carbonyl, hydroxyl and epoxy groups were observed in the spectra of GO suggesting the successful oxidation of Graphite recovered from waste Zn–C batteries.

However, the FTIR spectra of GO after CIP adsorption (GO-CIP) shows some changes in peak intensities. After accumulation of CIP on GO, the intensities of some peaks were shifted, disappeared and some peaks were more intense which indicates that oxygenated functional groups of GO may be involved in binding of CIP [80].

### 3.4 Raman analysis

Raman spectroscopy is an important way in characterizing carbonaceous materials, this is mostly due to the intense Raman peaks from carbon-carbon double bonds. Fig. 2(b) shows the Raman spectra of graphite powder (recovered from a Zn–C battery) and GO, two prominent peaks D and G were observed for both graphite powder and GO. The D and G peaks for graphite powder observed at 1355 cm^−1^ and 1581 cm^−1^, respectively. And the D and G peaks for GO observed at 1358 cm^−1^ and 1594 cm^−1^, respectively. This appearance of D and G peaks in Raman spectra is very typical for GO. The D peak is associated with the breathing modes of sp^2^ rings which are activated by defects in the structure, therefore, indicates the disorder [81,82]. The G peak is associated with the doubly degenerate E_2g_ phonons [62,83]. The ratio of intensities of the two peaks (I_D_/I_G_) reflects the extent of defects and quality of GO. The value of I_D_/I_G_ for GO (0.96) was found to be greater than that for Graphite (0.72), which might correspond to the incorporation of oxygenated groups that lead to defects in the structure.

### 3.5 BET analysis

The determination of surface area and pore distribution is crucial to understanding the adsorption property of any adsorbent. The larger the surface area and pore volume of a material, the more efficient it is as an adsorbent. The specific surface area, average pore width, and total pore volume were measured from the N_2_ adsorption-desorption isotherm by BET (Brunauer–Emmett–Teller) analysis (shown in Fig. 2(c and d)), which are 61.083426 m^2^ g^−1^, 3.84 nm, and 0.0926 cm^3^ g^−1^, respectively. Similar BET results have been shown in studies that used commercial graphite powder to prepare GO [84].

### 3.6 Adsorption studies

#### 3.6.1 Effect of pH

**Fig. 3:**
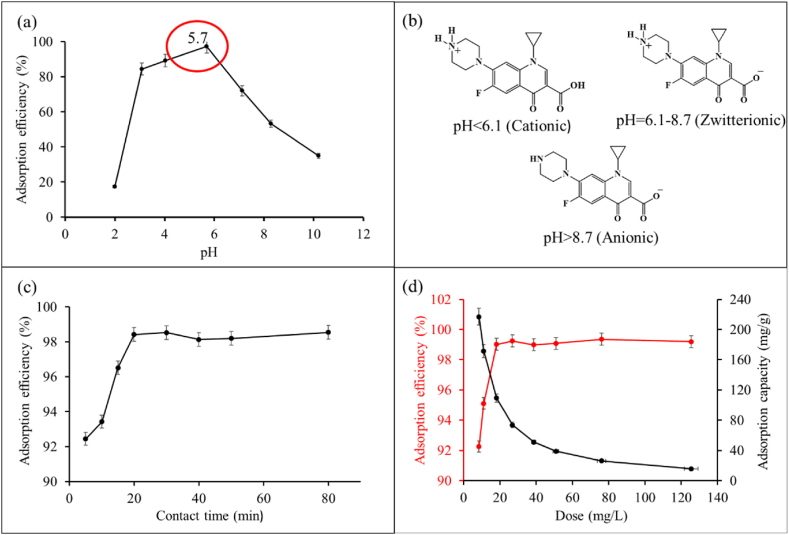
Effect of pH (a) ($C_{o}$ = 2.0 ppm, GO = 20 mg L^⁻1^, t = 20 min, shaking = 230 rpm, T = 30 °C), forms of CIP at different pH (b), effect of contact time (c) ($C_{o}$ = 2.0 ppm, GO = 20 mg L^⁻1^, pH = 5.7, shaking = 230 rpm, T = 30 °C), and effect of adsorbent dose (d) ($C_{o}$ = 2.0 ppm, pH = 5.7, t = 20 min, shaking = 230 rpm, T = 30 °C).

In an aqueous solution, pH plays an important role in the adsorptive removal of CIP onto GO as the electrostatic nature of both the CIP and the surface of GO depends on it. Depending on pH, CIP can exist as positively charged, zwitterion, and negatively charged species, it shows two pKa values (pKa1 = 6.1 and pKa2 = 8.7) [85] (Fig. 3(b)). So the interaction of CIP on the surface of GO could depend on solution pH. To explore the effect of pH on the adsorption process, changes in the adsorption efficiencies (%) of CIP were observed at pH ranges 2 to 10, as given in Fig. 3(a). Generally, electrostatic interaction is primarily considered when evaluating the impact of pH on removal efficiency, as this mechanism is commonly used to understand the adsorption of species from aqueous media [86]. The point of zero charge (pHzpc) of GO was measured pH 2.0 (Fig. S1). The surface charge of GO is positive at pH < pHzpc and negative at pH > pHzpc. At a pH below the pHzpc, the removal percentage of CIP will be decreased due to electrostatic repulsion between CIP and the GO surface. With the increase in pH of the solution, the surface of GO deprotonates and provides negatively charged active sites for the adsorption of cationic CIP, leading to maximum adsorption efficiency at pH 5.7> pHpzc. Above pH 5.7, adsorption efficiency continued to decrease until pH 10 due to zwitterionic and anionic CIP experience repulsive force from the deprotonated surface of GO.

#### 3.6.2 Effect of contact time

Contact time is a key parameter to evaluate the effectiveness of the removal process in a wastewater treatment plant. To study the effect of contact time, adsorption efficiencies (%) were observed at different durations of shaking (Fig. 3(c)). In the first 20 min, the adsorption efficiency sharply increased to a maximum with time, and active sites were successively occupied as the contact time was increased until all the active sites are saturated. Later, adsorption efficiency became steady with time, achieving adsorption equilibrium. 20 min of shaking was considered equilibrium time and maintained in isotherm and kinetic studies.

#### 3.6.3 Effect of adsorbent dose

Knowledge of the optimal adsorbent dose is crucial for the proper use of adsorbent. Adsorption efficiencies (%) at different adsorbent doses were observed by batch experiments (Fig. 3(d)). A sharp increase in adsorption efficiency was observed with the increase in adsorbent dose from 8.5 mg L^−1^ to 20 mg L^−1^; higher adsorbent dose serves more active sites for adsorption to take place, hence the increase in adsorption efficiency was observed. Further increase in adsorbent dose accompanied by no change in adsorption efficiency indicated the establishment of adsorption equilibrium. In another view, adsorption capacity decreases with the increase in adsorbent dose, this is because at higher adsorbent dose the surface area is less effectively used by the adsorbate, i.e., the number of unoccupied active sites increases with the increase in adsorbent dose. Interaction between the adsorbent particles might also play a role in the decline of adsorption capacity.

Judging the maximum removal of CIP and proper utilization of adsorbent, 20 mg L^−1^ was considered to be optimal adsorbent dose for this study.

#### 3.6.4 Isotherms

Isotherm models can explain the mechanism of adsorption, the capacity of the adsorbent, and nature of active sites. To find the best isotherm model that describes the adsorption process, experimental data were used to fit the Langmuir, Freundlich, Temkin, and Dubinin–Radushkevich (D-R) isotherm models. Equations (4), (5), (6), (7) express the linearized forms of the Langmuir, Freundlich, Temkin, and D-R isotherm models, respectively [87].

(4) $$\frac{C_{e}}{q_{e}}=\frac{C_{e}}{q_{m}}+\frac{1}{K_{L}q_{m}}$$

(5) $$\ln\;q_{e}=\ln\;K_{F}+\frac{1}{n}\ln\;C_{e}$$

(6) $$q_{e}=\frac{RT}{b}\ln\;K_{T}+\frac{RT}{b}\ln\;C_{e}$$

(7) $$\ln\;q_{e}=\ln\;Q_{m}-K_{E}{e_{p}}^{2}$$

Here, $C_{e}$ denotes the concentration of CIP at equilibrium (mg L⁻^1^); $q_{e}$ denotes adsorption capacity at equilibrium (mg g⁻^1^); $q_{m}$ is defined as the maximum adsorption capacity of GO at monolayer coverage (mg g⁻^1^); $K_{L}$ denotes Langmuir adsorption constant related to the affinity of binding sites (L g⁻^1^). $K_{F}$ represents Freundlich adsorption constant which is related to adsorption capacity (mg^1^⁻^1/n^L⁻^1/n^ g⁻^1^); n is a constant that refers to adsorption intensity. R stands for universal gas constant (J K⁻^1^mol⁻^1^); T is temperature (K); $K_{T}$ represents equilibrium binding constant (L g⁻^1^); b is a constant that refers to the heat of adsorption (J mol⁻^1^). $Q_{m}$ is a constant defined as maximum adsorption capacity (mg g⁻^1^); $K_{E}$ (mol^2^ kJ⁻^2^) is a constant that is connected to mean adsorption energy E (kJ mol⁻^1^) by equation (8); $e_{p}$ indicates Polanyi potential and is expressed by equation (9) (kJ mol⁻^1^).

(8) $$E=\frac{1}{\sqrt{2K_{E}}}$$

(9) $$e_{p}=RT\;\ln\left(1+\frac{1}{C_{e}}\right)$$

Equations (10), (11)) are the non-linear form of the Langmuir and Freundlich isotherm equations, respectively.

(10) $$q_{e}=\frac{q_{m}K_{L}C_{e}}{1+K_{L}C_{e}}$$

(11) $$q_{e}=K_{F}{\left(C_{e}\right)}^{\frac{1}{n}}$$

**Fig. 4:**
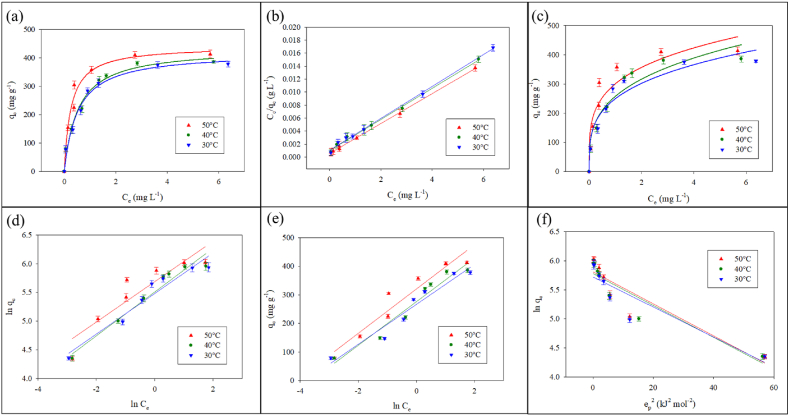
Non-linear Langmuir isotherms (a), Linearized Langmuir isotherms (b), Non-linear Freundlich isotherms (c), Linearized Freundlich isotherms (d), Temkin isotherm (e) and D-R isotherm models (f) (pH = 5.7, GO = 20 mg L^−1^, t = 20 min, shaking = 230 rpm, T = 30, 40 & 50 °C).

Parameters obtained from the fitting of these isotherm models (shown in Fig. 4(a–f)) are given in Table 1. Among the four isotherm models, Langmuir isotherm fitted best at the studied temperature (30°C–50°C) with the experimental data (R^2^ > 0.98). Based on the assumptions of the Langmuir isotherms, surface of graphene oxide provides energetically equivalent active sites for the formation of a monolayer of CIP, and the heat of adsorption does not depend on surface coverage [88,89]. Non-linear fitting was recommended in several studies to explain the parameters of the Langmuir and Freundlich isotherms [90,91]. From the non-linear Langmuir isotherm model, The maximum adsorption capacities of GO for CIP were found to be 419.62 mg g^−1^, 434.56 mg g^−1^ and 441.44 mg g^−1^ at temperature of 30 °C, 40 °C, and 50 °C, respectively.

**Table 1:** Parameters from isotherm model.

Isotherm model	Curve fitting	Parameters		30 °C	40 °C	50 °C
Langmuir	Linear	$q_{m}$	(mg g⁻^1^)	400.00	416.67	434.78
		$K_{L}$	(L mg⁻^1^)	2.5000	2.3999	3.8723
		R^2^		0.9966	0.9959	0.9989
	Non-Linear	$q_{m}$	(mg g⁻^1^)	419.62	434.56	441.44
		$K_{L}$	(L mg⁻^1^)	1.9385	1.8854	3.8723
		R^2^		0.9800	0.9844	0.9814
Freundlich	Linear	$K_{F}$	(mg^1^⁻^1/n^ L⁻^1/n^ g⁻^1^)	240.13	246.56	295.15
		n		2.8361	2.6240	2.8506
		R^2^		0.9370	0.9551	0.8395
	Non-Linear	$K_{F}$	(mg^1^⁻^1/n^ L⁻^1/n^ g⁻^1^)	249.29	257.48	300.39
		n		3.6114	3.3636	4.0371
		R^2^		0.9438	0.9443	0.9018
Temkin	Linear	$K_{T}$	(L g⁻^1^)	45.22	37.23	62.09
		b	(J mol⁻^1^)	35.99	34.05	34.57
		R^2^		0.9433	0.9505	0.9255
Dubinin–Radushkevich	Linear	$Q_{m}$	(mg g⁻^1^)	305.03	323.89	333.52
		$K_{E}$	(mol^2^ kJ⁻^2^)	0.0258	0.0274	0.0275
		E	(mol^2^ kJ⁻^2^)	4.4022	4.2718	4.2640
		R^2^		0.8354	0.8733	0.8372

Table 2 gives a comparison of the maximum adsorption capacities ($q_{m})$ of different adsorbents and the equilibrium time for the adsorptive removal of CIP. As compared to other previous studies, the adsorbent prepared in this study had a comparatively high adsorption capacity for CIP of 419.62 mg g^−1^. Besides, the time required to reach equilibrium was the quickest in comparison with most of the previous studies. Generally, the major dependent factors for adsorption by adsorbent are as follows: 1) nature of adsorbent: functional groups, surface area, pore distribution, pore volume, particle size and polarity; (2) nature of adsorbate: molecular size, functional groups, and polarity; and (4) adsorption conditions: pH, temperature, adsorbent dosage, contact time, and the initial adsorbate concentration. In this study we have found maximum adsorption capacity within very short time due to different types of interaction involved for adsorption process. At optimal condition there are different types of interaction possible between GO and CIP such as H bonding, electrostatic interaction. Moreover, if we see with the structural view, CIP is enriched with pi electrons which intensify the adsorption capacity by pi-pi interaction more than from previous studies. Therefore, considering the adsorption capacity and equilibrium time, GO derived from Zn–C battery may be considered as promising low cost and environmentally friendly adsorbent to remove CIP from aqueous media.

**Table 2:** Comparison of Maximum adsorption capacities of different adsorbents and Equilibrium 467 time for adsorption of CIP.

Adsorbent	Maximum adsorption capacity, $q_{m}$ (mg g⁻^1^)	Equilibrium time (minutes)	Temperature (°C)	pH	Reference
Titanate nanotubes	153.90	2880	25	5	[92]
Magnetic sludge biochar	74.2	1440	25	NA	[93]
*Prosopis juliflora* wood	250	1440	25	4	[94]
Graphene oxide/calcium alginate composite	66.25	1440	25	6.1	[95]
Graphene hydrogel	235.6	720	25	NA	[96]
Protein-Modified Nanosilica	85	120	25	10	[97]
**GO (prepared from waste Zn–C battery)**	**419.62**	**20**	**30**	**5.7**	**Present study**

#### 3.6.5 Thermodynamics study

**Fig. 5:**
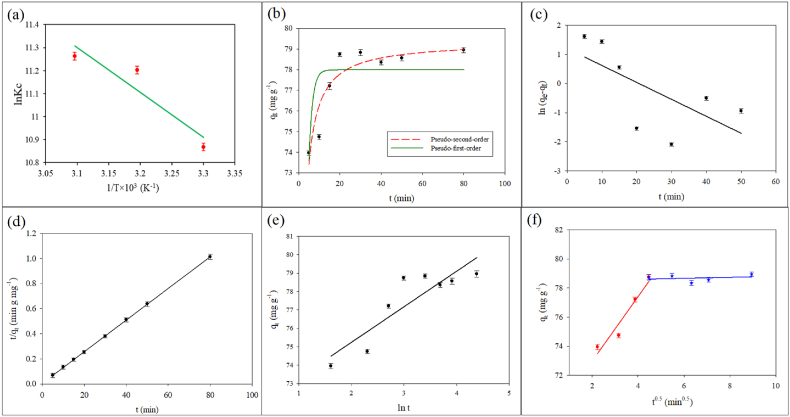
Plot for the determination of thermodynamic parameters models (a), Non-linear pseudo-first-order and pseudo-second-order kinetic models (b) and Linearized pseudo-first-order (c), pseudo-second-order (d), Elovich (e) intraparticle diffusion (f) and plot for the determination of thermodynamic parameters models (f), ($C_{o}$ = 2.0 ppm, GO = 20 mg L^−1^, pH = 5.7, shaking = 230 rpm, T = 30 °C).

Thermodynamics studies provide insight into the viability and mechanism of the adsorption. Experimental data were plotted in Fig. 5(a) using equation (12), and the change of enthalpy (ΔH) and entropy (ΔS) were determined from that plot. Equation (13) was used to calculate the change in Gibb's free energy (ΔG) at different temperatures.

(12) $$\ln\;K_{c}=-\frac{\Delta H}{RT}+\frac{\Delta S}{R}$$

(13) ΔG=ΔH−TΔS

Here, R refers to the universal gas constant (J K⁻^1^ mol⁻^1^). T is temperature (K). Thermodynamic equilibrium constant (K_c_) was calculated according to the reported method as ${lnK}_{c}=\frac{q_{e}}{C_{e}}\times 1000$ [86]

Table 3 shows the estimated thermodynamic parameters. The enthalpy change (ΔH) was positve, which indicates heat is absorbed during adsorption, and an increase in temperature favors the adsorption. The positive value of entropy change (ΔS) implies that the randomness within the system increased by adsorption. It can be envisaged from the positive values of ΔH and ΔS that the CIP molecules might have undergone dissociative chemisorption on the surface of GO [98,99]. The negative values of Gibb's free energy (ΔG) signifies that the adsorption was an spontaneous process over the studied temperature range. And increase in temperature caused larger negative ΔG values suggesting higher temperature favors the adsorption.

**Table 3:** Thermodynamic parameters.

T (K)	ΔH (kJ mol⁻^1^)	ΔS (kJ mol⁻^1^ K⁻^1^)	ΔG (kJ mol⁻^1^)
303	16.18	0.144	−27.48
313			−28.92
323			−30.37

#### 3.6.6 Kinetics study

Kinetic studies provide insight into the mechanism of adsorption. For kinetic studies, experimental data were fitted to Lagergren's pseudo-first-order, Ho's pseudo-second-order, Elovich, and intraparticle diffusion models. Equations (14), (15), (16), (17) demonstrate the linear forms of pseudo-first-order, pseudo-second-order, Elovich, and intraparticle diffusion models.

(14) $$\ln\left(q_{e}-q_{t}\right)=\ln\;q_{e}-k_{1}t$$

(15) $$\frac{t}{q_{t}}=\frac{1}{k_{2}q_{e}^{2}}+\frac{t}{q_{e}}$$

(16) $$q_{t}=\frac{\ln\;a_{e}b_{e}}{b_{e}}+\frac{1}{b_{e}}\ln\;t$$

(17) $$q_{t}=k_{i}t^{0.5}+C_{i}$$

Equations (18), (19)) represent the pseudo-first-order and pseudo-second-order models' non-linear forms.

(18) $$q_{t}=q_{e}\left(1-{\mathrm{e}}^{-k_{1}t}\right)$$

(19) $$q_{t}=\frac{k_{2}q_{e}^{2}t}{1+k_{2}q_{e}t}$$

Here, $q_{e}$ denotes adsorption capacity at equilibrium (mg g⁻^1^); $q_{t}$ denotes adsorption capacity at time t (mg g⁻^1^); $k_{1}$ is rate constant of pseudo-first-order model (g mg⁻^1^ min⁻^1^); $k_{2}$ is rate constant of the pseudo-second-order model (g mg⁻^1^ min⁻^1^); $a_{e}$ indicates initial adsorption rate (mg g⁻^1^ min⁻^1^); $b_{e}$ signifies the extent of surface coverage and activation energy for chemisorption (g mg⁻^1^); $k_{i}$ is rate constant from intraparticle diffusion model (mg g⁻^1^ min⁻^0.5^); and $C_{i}$ is a constant which signifies boundary layer thickness (mg g⁻^1^). The linear fitting can sometimes produce erroneous result, therefore both the linear and non-linear fitting of the kinetic models were studied [100]. The parameters obtained from the kinetic models are given in Table 4.

**Table 4:** Parameters from kinetic model.

Kinetic model	Curve fitting	Parameters		CIP (2 ppm)	
Pseudo-first order	Linear	$q_{e}$	(mg g⁻^1^)	3.26	
		$k_{1}$	(g mg⁻^1^ min⁻^1^)	0.0581	
		R^2^		0.4363	
	Non-Linear	$q_{e}$	(mg g⁻^1^)	77.99	
		$k_{1}$	(g mg⁻^1^ min⁻^1^)	0.5756	
		R^2^		0.5500	
Pseudo-second order	Linear	$q_{e}$	(mg g⁻^1^)	79.36	
		$k_{2}$	(g mg⁻^1^ min⁻^1^)	0.0331	
		R^2^		1.0000	
	Non-linear	$q_{e}$	(mg g⁻^1^)	79.36	
		$k_{2}$	(g mg⁻^1^ min⁻^1^)	0.0311	
		R^2^		0.8584	
Elovich	Linear	$a_{e}$	(mg g⁻^1^ min⁻^1^)	2.28 × 10^16^	
		$b_{e}$	(g mg⁻^1^)	0.5185	
		R^2^		0.7806	
Intraparticle diffusion	Linear	$k_{i}$	(mg g⁻^1^ min⁻^0.5^)	Line-1	2.2176
				Line-2	0.0337
		R^2^		Line-1	0.9352
				Line-2	0.0584
		$C_{i}$	(mg g⁻^1^)	Line-1	68.53
				Line-2	78.46

Among the four kinetic models (shown in Fig. 5(b–f)), pseudo-second order fitted best against the experimental data (R^2^ > 0.85). Besides, $q_{e}$ value (79.36 mg g^−1^) calculated from the pseudo-second order model matched best with the experimental $q_{e}$ value (78.82 mg g^−1^). They altogether suggest that, it is best to describe the kinetics of the adsorption process by pseudo-second order model and the adsorption may undergo a chemisorption mechanism [101,102].

#### 3.6.7 Adsorption-desorption mechanism

**Fig. 6:**
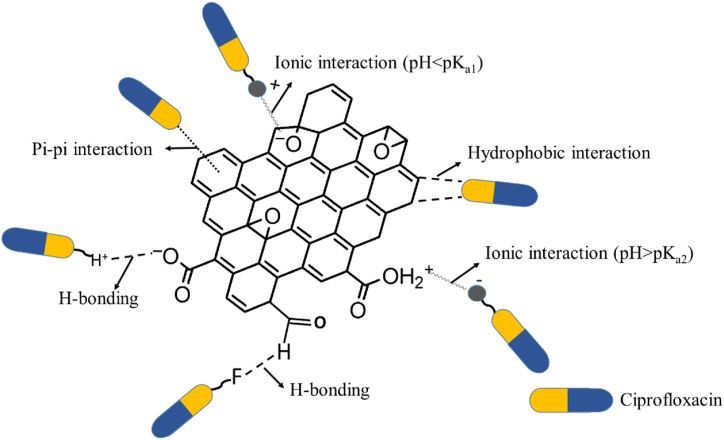
Probable interaction of CIP with the surface of Graphene oxide.

Both the chemical and physical nature of the adsorbent and adsorbate, and the pH of the medium contribute to the mechanism of adsorption. The adsorption efficiency of ciprofloxacin (CIP) on graphene oxide (GO, pH_ZPC_ = 2.0) varies with pH. The probable mechanism for the adsorption of CIP onto GO is shown in Fig. 6. At pH < 2.0 (pH_ZPC_ of GO) the GO surface is neutral and CIP exists predominantly as a cationic (positively charged) species. The electrostatic interaction is minimal due to the neutral GO surface, but the pi systems of the CIP and GO participate in a pi-pi interaction, that assists adsorption at all pH [103]. H-bonding can occur from both sides, from the electronegative atoms such as F and O of CIP to partially electropositive H atoms attached to functional groups of GO, and vice versa [104]. the pH value is higher than the pHpzc, the adsorbent surface will be negative. This results in strong electrostatic attraction between the negatively charged GO and positively charged CIP, along with enhanced π-π interactions due to this electrostatic attraction [104]. This phenomenon is leading to an increase in the adsorption percentage of cationic adsorbates like CIP on GO. The electrostatic interaction occurred between the negative surface charges of GO and the protonated amine groups of CIP. In this study, pH 5.7 was found to be optimum for favorable ionic interactions that gave rise to the highest adsorption efficiency. The hydrophobic interaction may also contribute to the adsorption [105].

#### 3.6.8 DFT study for adsorption mechanism

**Fig. 7:**
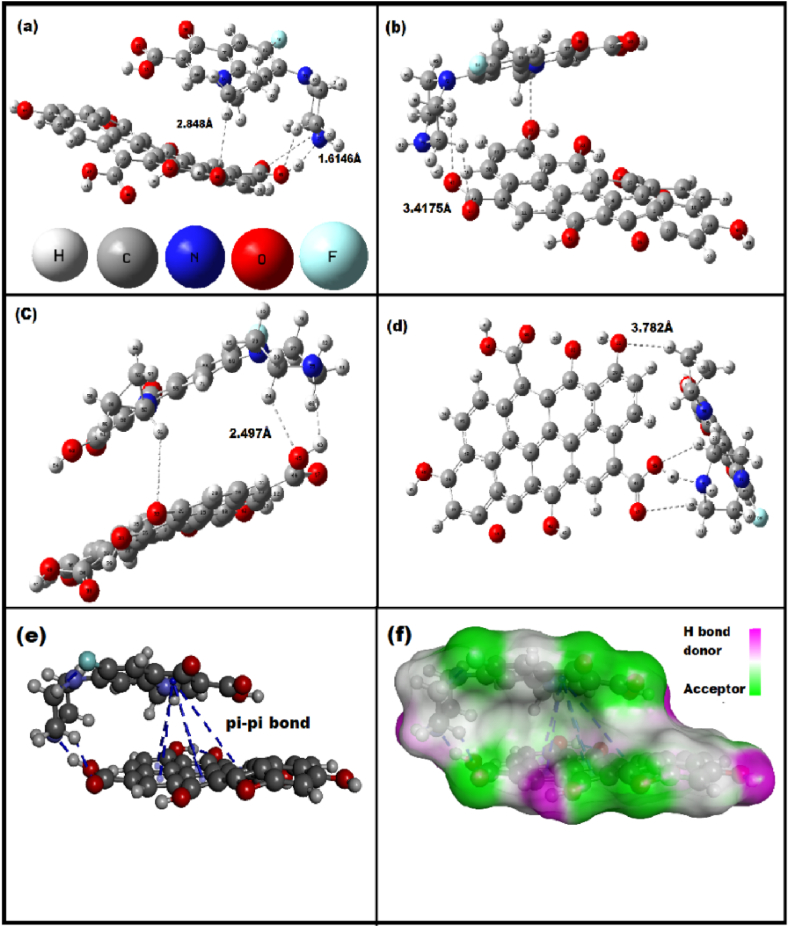
Optimization structure of GO-CIP complex after adsorption process (a–d) and (e–f) are pi-pi interaction and H bonding.

In this study, we have used Density function theory (DFT) to study the adsorption mechanism at the electronic level. All the structure was optimized by using DFT and the 6-311G basis set [106]. The enthalpy was determined from the optimized structure and the value of theoretical enthalpy (ΔH) is 16.939 kJ/mol. Experimental enthalpy was obtained at 16.18 kJ/mol, which is very close to theoretical enthalpy. The hydrogen bonds were observed between GO and CIP, which the H46 atom of the carboxylic group of GO and the N75 atom of the CIP molecule, with H⋯N distance being 1.614 Å, and another was formed by the O47 atom of –COOH group of GO and the H80 atom of the CIP, with OH O⋯H distance being 2.887 Å. Furthermore, the H89 and H91 atom of the CIP molecule was connected by the hydrogen bond with the O32 atom of the hydroxyl group of the GO respectively O32⋯H89 (2.848 Å) and O32⋯H91 (3.628 Å) (Fig. 7(a–d)). Increase and decrease the bond length, angle, and degree of HOMO and LUMO in order to promote the electrostatic, Pi-Pi interaction between CIP and GO [107]. Additionally, pi-pi and electrostatic interaction between GO and CIP have been observed in the discovery studio, where the optimized log file was converted to Pdb (Fig. 7(e and f). Above, all types in types inter action support the experimental data [108]. Above all, all interactions are consistent with the experimental data. All DFT calculations were explained in detail in supplementary sections (1,2,3,4 and 5).

#### 3.6.9 Stability and reusability

**Fig. 8:**
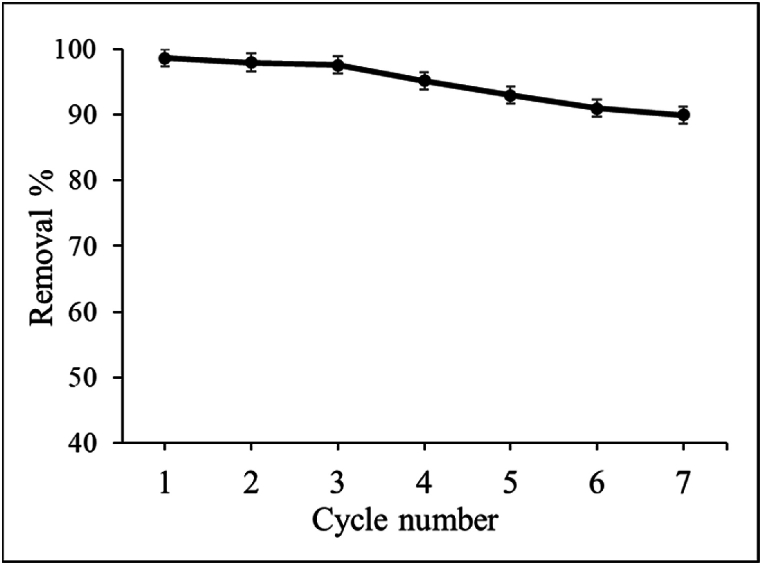
Stability and reusability test for GO adsorbent.

Stability and reusability are the most crucial factors for any adsorbent and these factors are highly desirable for both environmental and financial reasons. As a result, throughout the course of seven cycles, the adsorbent's stability and reusability were assessed. In this work, we have used methanol for the regeneration of the adsorbent. After adsorption of CIP, the GO was effectively re-generated by the treatment with methanol. The change of the removal percentage was represented in Fig. 8. As shown in Fig. 8, the adsorbent was still able to remove 92% of the CIP after seven consecutive cycles. This proved that the GO prepared from waste Zn–C battery has superior stability and reusability.

## 4 Conclusion

In the preparation of GO, carbon rods from waste Zn–C batteries were successfully utilized, which set up facilities for low-cost GO preparation, solid waste management, and the recycling of valuable graphite. The successful inclusion of various oxygen-containing functional groups and the porous, wrinkled, and rough texture of the surface of the prepared GO rendered many active sites and a large surface area for the adsorption of CIP onto GO. As a result, the CIP adsorption on GO was achieved with a minimal adsorbent dosage of 20 g/L, and the maximum adsorption (99.0%) was reached in only 20 min while the initial concentration of CIP was 2.0 ppm.The experimental data of the adsorption fitted Langmuir isotherm model very well, and the maximum adsorption capacity was calculated to be 419.62 mg g^−1^ at 30 °C. Moreover, the adsorption was spontaneous over 30–50 °C. The results show that Zn–C battery generated GO has good adsorption properties for CIP in relatively short time and the adsorption process lead by electrostatic attractions, H-bonding interaction, and pi-pi interaction, which is consistent with density functional theory. Considering the results, it can be said that, this work provides a fast, efficient, and viable method for preventing the pollutant CIP from entering the water bodies. This research also may encourage researchers in the future to employ this adsorbent in water treatment plants in an appropriate way.

## CRediT authorship contribution statement

**Sabina Yasmin:** Writing – review & editing, Writing – original draft, Supervision, Resources, Project administration, Methodology, Funding acquisition, Data curation, Conceptualization. **Md Golam Azam:** Writing – original draft, Formal analysis, Data curation. **Md Sanwar Hossain:** Writing – original draft, Formal analysis, Data curation. **Umme Sarmeen Akhtar:** Writing – review & editing, Data curation. **Md Humayun Kabir:** Writing – review & editing, Writing – original draft, Visualization, Supervision, Resources, Project administration, Methodology, Investigation, Funding acquisition, Data curation, Conceptualization.

## Declaration of competing interest

The authors declare no conflict of interest.
