# Supplementary material for: Efficient removal of ciprofloxacin from aqueous solution using Zn–C battery derived graphene oxide enhanced by hydrogen bonding, electrostatic and π-π interaction
Source: Heliyon. 2024 Jun 21;10(12):e33317. doi: 10.1016/j.heliyon.2024.e33317 (PMC11253669; doi:10.1016/j.heliyon.2024.e33317)
Supplement: Multimedia component 1 [file mmc1.docx]

Efficient Removal of Ciprofloxacin from Aqueous solution using Zn-C battery derived Graphene oxide enhanced by Hydrogen Bonding, Electrostatic and π-π Interaction

Sabina Yasmin*, Md. Golam Azam, Md. Sanwar Hossain, Md Humayun Kabir*

Institute of National Analytical Research and Service (INARS), Bangladesh Council of Scientific and Industrial Research (BCSIR), Dhanmondi, Dhaka-1205, Bangladesh

*Corresponding author e-mail: (Sabina Yasmin) [sabinayasmin@bcsir.gov.bd](mailto:sabinayasmin@bcsir.gov.bd%20)

*Corresponding author e-mail: (Md Humayun Kabir) [humayunkabir@bcsir.gov.bd](mailto:humayunkabir@bcsir.gov.bd)

**Computational method (section 1)**

**Geometry optimization graphene oxide, and ciprofloxacin (section 2)**

**DFT study for adsorption mechanism (section 3)**

**Molecular orbital analysis (section 4)**

**Enthalpy change analysis (5)**

**Table S1, Table S2, Table S3 and Table S4**

**Figure S1 and Figure S2**

**Section:1**

**Computational method:** Density Function Theory (DFT) is a powerful theoretical framework in quantum mechanics that is widely used in condensed matter physics, materials science, chemistry, and related fields [1][2]. It provides a way to describe and understand the electronic structure and properties of molecules, solids, and nanostructures. Quantum chemical computations were used to predict the equilibrium geometry of graphene oxide, ciprofloxacin, and the interaction of graphene Oxide and, ciprofloxacin, In this case, the DFT approach was employed to fully optimize the original molecular mechanics-established structures of graphene oxide, ciprofloxacin. The Becke three-parameter hybrid approach with LYP correlation (B3LYP) level of DFT and the 6-311G basis set was used to calculate the electrical properties and optimize the geometry for all atoms level of DFT and the 6-311G basis set [3]. Additionally, dipole moment, HOMO, and LUMO energy were measured in both vacuum and water solutions. Fig.S1 depicts the final optimized graphene oxide (GO) (a) and ciprofloxacin (b) and respectively. All calculation has been done by GaussView 5 software.

**Section:2**

**Geometry optimization graphene oxide, and ciprofloxacin:** Geometry optimization of graphene oxide (GO) and ciprofloxacin Figure S1 shows. In the GO, the bond angle in the hexagonal planar molecular geometry is approximate to 120 [4], [5]. In graphene oxide, one of the largest bond length is calculated as 1.8221 and assigned to O38-H39, while one of the smallest bond length is calculated as 0.9956 and assigned to O48–H49. The most of the bond angle of inter carbon atom 120º and C8-C9-C10, C8-C9-C15, C9-C8-C16 bond angles are 120.828, 120.529 and 120.99, which in indicate that triangular planar geometry of graphene oxide. The bond length and angle are shown in Table S2. In ciprofloxacin, one of the largest bond angles is calculated as 120.518 and assigned to C11–C8–9, while one of the smallest bond angles is calculated as 60.09 and assigned to C–C1–37 of ciprofloxacin. The surrounding three bond angles H31–N24–C25, H35–C22–N31 and H10–C9–N3 around the N atom are equal to 111.061,109.447 and 118.963, respectively, with an average of 113.157 in table S2 [4].


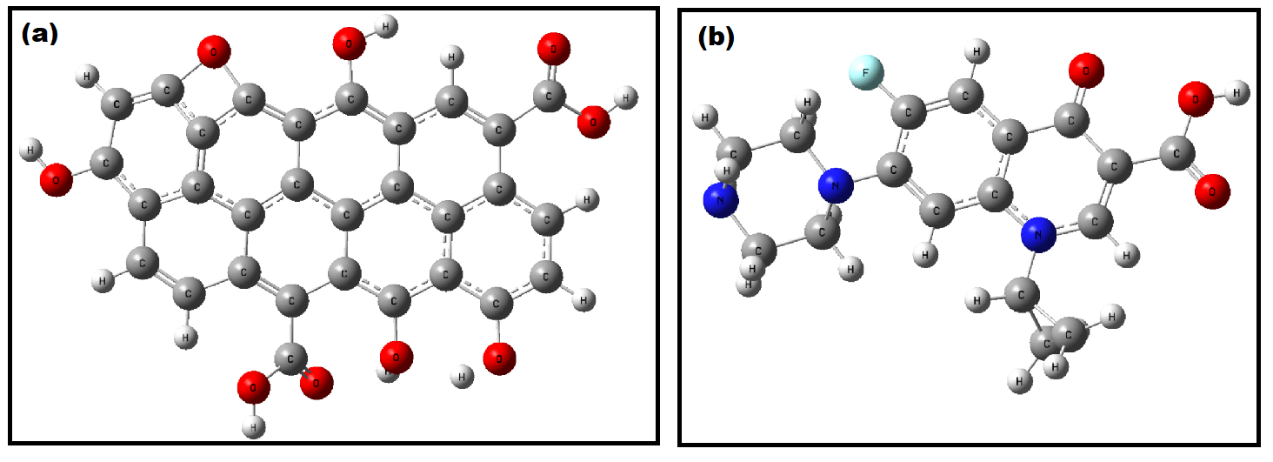


Figure. S1 Optimized structure of graphene oxide (a) and ciprofloxacin (b) at B3LYP/6-311G level of theory.

**Section 3**

**DFT study for adsorption mechanism:** DFT allows for a detailed analysis of the electronic structure of the adsorbate-surface system. By examining the electronic density of states, bond lengths, and bond strengths, researchers can gain insights into the nature of chemical bonding and orbital interactions involved in the adsorption process [1]. Furthermore, the contact energy values show that the ciprofloxacin molecule has a robust absorption process on the GO. The hydrogen bonds are observed at the GO edge for ciprofloxacin adsorption in the most stable configuration one is formed by the H46 at of carboxylic acid functional group of GO and the N75 atom of ciprofloxacin molecule, with H···N distance being 1.614 Å, and another is formed by the O45 atom of -COOH group of GO and the H80 atom of the CIP molecule, O···H distance being 3.2943Å. Furthermore, the hydroxyl group of the GO molecule is hydrogen connected to the GO edge, respectively O32···H89 (2.848Å) and O33···H89 (3.791 Å). It should be pointed out that the presence of more hydrogen bonding contacts results in the highest interaction energy of CIP molecule with GO (see Tables S1). Also, the shortest hydrogen bond at structure is formed by an oxygen atom of the carboxyl functional group of GO and the NH terminal of the CIP molecule.

| Name of Atom | Bond length (Å) |
| --- | --- |
| N75–H46 | 1.6146 |
| H82–O45 | 3.2943 |
| O47–H80 | 2.3919 |
| O47–H81 | 3.4175 |
| O45–H84 | 2.4197 |
| O45–H83 | 3.381 |
| O45-H82 | 3.305 |
| H32–O89 | 2.831 |
| O32–H91 | 3.848 |
| H89–O33 | 3.790 |
| O45-H46 | 1.053 |

TableS1. The calculated intermolecular distances (Å) between the adsorbed CIP and GO in the studied configurations

The lengths of the C44=O47, O45-C46, and C34-O40 bonds in the GO molecule are elongated to 1.214, 0.999, and 1.375Å from their original lengths of 1.251,1.061, and 1.377 Å, respectively, as a result of the formation of intermolecular hydrogen bonds at the most stable complex, according to an analysis of the structural parameters and these are caused by CIP's adsorption to GO. In the current instance, the more prominent construct of C74-H84, C76-H80, and C62-O65 indicate that CIP has a stronger affinity to CIP. In CIP adsorption with GO, the bridging angles C1-C2-C3, C10-C7-O42, and O45-C44-O47 of GO increase 118.133,124.152, and 118.525º to 119.201, 124.828, and 121.556º (table S2). The degree of change in the HOMO-LUMO energy difference as a result of interaction can describe (section 2) the type and nature of bonding or interaction between GO and ciprofloxacin molecules (Fig.S2 and Table S2). Increase and decrease the bond length and angle in order to promote the electrostatic, Pi-Pi interaction between CIP and GO.

TableS2. Bond length and bond angle of GO and CIP before and after adsorption

| Number of molecule | Number of atom | Bond length Before adsorption | Bond length After adsorption(Å) |
| --- | --- | --- | --- |
| GO and complex of GO-CIP | C2-O51 | 1.478 | 1.535 |
|  | C3-C4 | 1.478 | 1.484 |
|  | C27-H28 | 1.090 | 1.084 |
|  | O33-39H | 1.021 | 1.020 |
|  | C34-C37 | 1.473 | 1.454 |
|  | C34-O38 | 1.220 | 1.270 |
|  | C34-O40 | 1.375 | 1.377 |
|  | C36-H50 | 1.096 | 1.076 |
|  | O38-H39 | 1.822 | 1.469 |
|  | O40-H41 | 0.999 | 0.982 |
|  | O42-H43 | 1.005 | 0.975 |
|  | C44-O45 | 1.374 | 1.349 |
|  | C44=O47 | 1.214 | 1.251 |
|  | O45-C46 | 0.999 | 1.061 |
|  | O48-49H | 0.996 | 0.976 |
| CIP, and the complex of GO-CIP | N75-H82 | 1.027 | 1.009 |
|  | C74-H84 | 1.117 | 1.090 |
|  | C76-H80 | 1.116 | 1.090 |
|  | C62-O65 | 1.207 | 1.216 |
| Name of molecule | Number of atom | Bond angle Before adsorption (degree) | Bond angle After adsorption(degree) |
| GO and complex of GO-CIP | C9-C8-C16 | 120.829 | 121.349 |
|  | C8-C9-C10 | 120.529 | 120.771 |
|  | C8-C9-C15 | 120.991 | 120.590 |
|  | C1-C2-C3 | 118.133 | 119.201 , |
|  | C3-C2-O51 | 148.375 | 146.871 |
|  | C4-C3-C7 | 117.453 | 119.292 |
|  | C10-C7-O42 | 124.152 | 124.828 |
|  | C10-C11-H12 | 117.227 | 120.928 |
|  | C26-C16-C37 | 123.729 | 125.546 |
|  | C14-C20-H31 | 120.587 | 118.565 |
|  | C18-C24-O48 | 115.362 | 115.934 |
|  | C22-C24-O48 | 118.078 | 119.235 |
|  | C18-C25-H30 | 117.362 | 120.043 |
|  | C18-C25-H30 | 120.940 | 120.043 |
|  | C16-C26-C19 | 124.083 | 123.161 |
|  | C17-C37-C34 | 120.105 | 120.635 |
|  | C34-O40-C41 | 111.966 | 107.748 |
|  | C7-O42-C43 | 112.401 | 113.266 |
|  | C13-C44-O47 | 127.040 | 121.964 |
|  | O45-C44-O47 | 118.525 | 121.556 |
| CIP, and the complex of GO-CIP | O63-C63-O65 | 117.675 | 119.965 |
|  | C74-N75-C76 | 100.199 | 109.866 |
|  | C73-N72-C77 | 122.106 | 112.106 |

**Section: 4**

**Molecular orbital analysis:** The interaction between two interacting species takes place when the frontier molecular orbitals participate with each other, according to quantum mechanics. The degree of change in the HOMO-LUMO energy difference as a result of interaction can describe the type and nature of bonding or interaction between GO and CIP molecules. The energy of a chemical species is also connected to its reactivity. The higher the energy associated with HOMO, the greater will be its reactivity and vice versa stability [6]. TableS3 shows the band gaps in terms of the difference between GO's HOMO and LUMO. TableS1 includes the energies of HOMO and LUMO (EHOMO and ELUMO) before and after complexation, as well as the variation in energy difference caused in HOMO (EHOMO) by complexation (Fig.S2). The difference in energies of HOMO and LUMO, or the HOMO-LUMO (H-L) gap or bandgap, can be used to calculate a chemical species' electrical conductivity. Moving the band gap analysis, the GO-CIP complex is more stable since the electron can readily migrate due to the complex's small band.


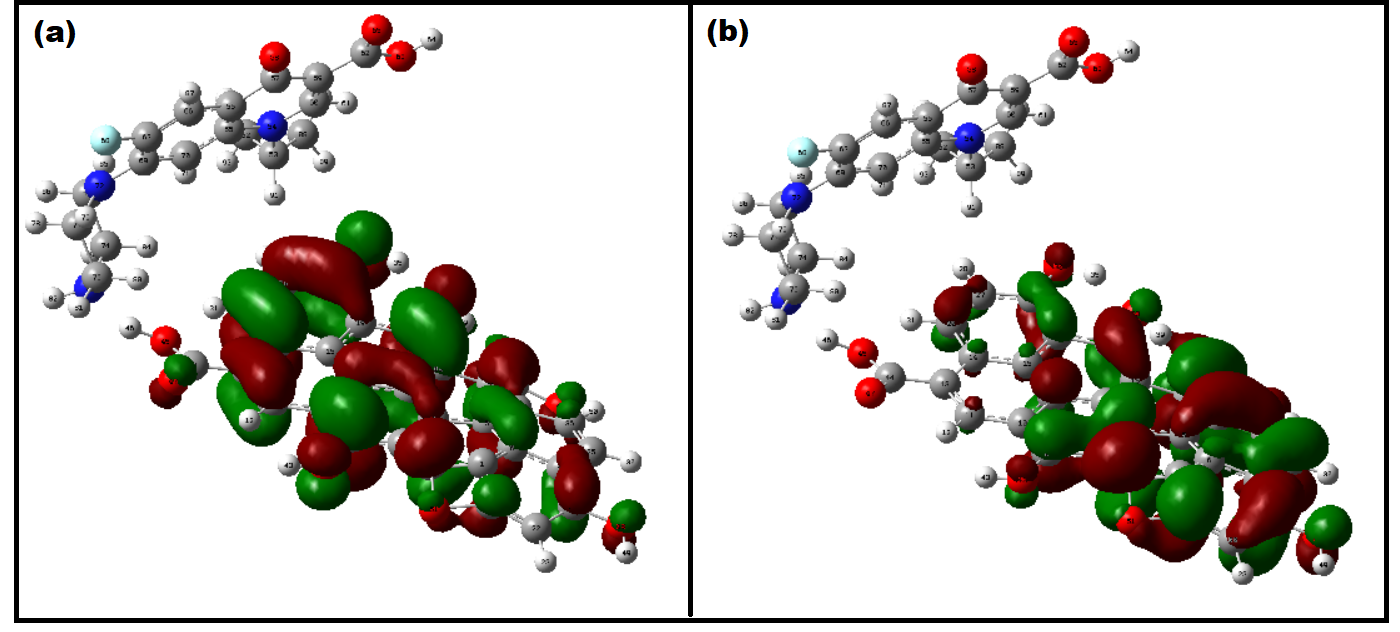


Figure. S2Topology of HOMO and LUMO orbitals of graphene oxide, and CIP calculated at B3LYP/6-311G level of theory.

TableS3.The values of the highest occupied molecular orbital energies (EHOMO) and the lowest unoccupied molecular orbital energies (ELUMO), Band gap

| Name Component | HOMO(eV) | LUMO(eV) | Band gap(eV) |
| --- | --- | --- | --- |
| GO | -7.741 | -2.429 | -5.31 |
| CIP | -5.662 | -1.688 | -3.97 |
| GO-CIP | -4.787 | -3.294 | -1.49 |

**Section:5**

**Enthalpy change analysis:** Understanding the enthalpy change in the adsorption process is essential for predicting and controlling the thermodynamics and energetics of adsorption systems. Experimental techniques such as calorimetry and computational modeling approaches are commonly employed to measure and estimate the enthalpy change associated with adsorption processes. Theoretical enthalpy is 16.939 KJ/mole and positive value indicates that endothermic reaction (TableS4). The experimental enthalpy, which is 16.18 KJ/mole and nearly identical to the theoretical enthalpy that has been obtained. In these interaction and enthalpy data analysis we understood that theoretical very is correlated to the experimental result.

| Name of Molecule | Enthalpy (KJ/mole) | Dipole moment(Debye) | Enthalpy (KJ/mole) |
| --- | --- | --- | --- |
| Graphene Oxide (GO) | 996.3398 | 5.6803 | 16.93913 |
| Ciprofloxacin (CIP) | 939.9889 | 8.585 |  |
| GO-CIP Complex | 1953.268 | 10.3546 |  |

Table S4. Enthalpy and Dipole moment

**Section 6:**





Figure S1. Plot showing point of zero charge of GO under different pH conditions

References

[1] W. Kohn, A. D. Becke, and R. G. Parr, ‘Density Functional Theory of Electronic Structure’, *The Journal of Physical Chemistry*, vol. 100, no. 31, pp. 12974–12980, Jan. 1996, doi: 10.1021/jp960669l.

[2] B. B. Laird, R. B. Ross, and T. Ziegler, ‘Density-Functional Methods in Chemistry : An Overview Density-functional theory ( DFT ), in its various forms , has become an important research tool for chemists , physicists and materials scientists . Its development in recent years has proceeded along ’, pp. 1–17, 1996.

[3] J. Tirado-Rives and W. Jorgensen, ‘Performance of B3LYP Density Functional Methods for a Large Set of Organic Molecules’, vol. 4, pp. 297–306, Feb. 2008, doi: 10.1021/ct700248k.

[4] D. W. Boukhvalov, ‘DFT modeling of the covalent functionalization of graphene: from ideal to realistic models’, *RSC Advances*, vol. 3, no. 20, pp. 7150–7159, 2013, doi: 10.1039/C3RA23372C.

[5] D. W. Boukhvalov and M. I. Katsnelson, ‘Modeling of graphite oxide.’, *Journal of the American Chemical Society*, vol. 130, no. 32, pp. 10697–10701, Aug. 2008, doi: 10.1021/ja8021686.

[6] S. Chen, S. Scheiner, T. Kar, and U. Adhikari, ‘Theoretical study on relationship between structure of mercapto-triazole derivatives and inhibition performance’, *International Journal of Electrochemical Science*, vol. 7, no. 8, pp. 7128–7139, 2012.
